# Predicting Powder Blend Flowability from Individual Constituent Properties Using Machine Learning

**DOI:** 10.1007/s11095-025-03855-x

**Published:** 2025-04-17

**Authors:** Anna Owasit, Siddharth Tripathi, Rajesh Davé, Joshua Young

**Affiliations:** Otto H. York Department of Chemical and Materials Engineering, New Jersey Institute of Technology, 138 Warren St, Newark, NJ 07103 USA

**Keywords:** Dry coating, Flowability, Machine learning (ML), Pharmaceutical powder blends, Powder flow prediction

## Abstract

**Purpose:**

Predicting powder blend flowability is necessary for pharmaceutical manufacturing but challenging and resource-intensive. The purpose was to develop machine learning (ML) models to help predict flowability across multiple flow categories, identify key predictive features, and arrive at formulations with improved flow properties.

**Methods:**

A dataset of 410 blends, composed of 9 active pharmaceutical ingredients (APIs) and 18 excipients with varying silica dry-coating parameters, was analyzed. Supervised ML models were trained to predict various flowability categories (very cohesive, cohesive, semi-cohesive, well-flowing, and free-flowing). Particle size, morphology, surface properties, and coating parameters were used as features. Classification algorithms, including Random Forest (RF) and Extreme Gradient Boosting (XGBoost), were evaluated. Unsupervised clustering identified natural groupings within flowability data.

**Results:**

The best-performing models achieved up to 85% accuracy for predicting flowability regimes of individual components and 87% for blends. Individual components generally showed higher accuracy than blends, except in the uncoated scenario with 2 flow regimes, where blends outperformed with 94.67%. SHapley Additive exPlanations (SHAP) and Feature Importance analysis indicated dry coating parameters as the most influential factors, followed by particle size and morphology. ML models effectively identified category transitions between flow regimes, offering insights into blend optimization.

**Conclusion:**

Integrating ML with mechanistic approaches effectively predicted powder blend flowability across diverse categories and elucidated feature-property relationships. These outcomes can facilitate the rational design of blends having enhanced flow properties at reduced experimental effort through judiciously selected dry coating of a blend constituent; making this approach promising for advancing pharmaceutical process and product development.

## Introduction

Adequate flowability of pharmaceutical powders is a critical factor in ensuring the efficiency and consistency of various pharmaceutical manufacturing processes, particularly in tableting. The flow behavior directly affects tablet weight uniformity and active pharmaceutical ingredient (API) distribution [1, 2]. Several particle properties, including particle size distributions, surface energy, density, morphology, and surface roughness, play a crucial role in determining powder flow characteristics [1, 3]. Moreover, various interparticle forces, such as van der Waals forces, chemical bonding, electrostatic interactions, and liquid bridging due to adsorbed moisture, significantly influence powder flow properties. These forces can cause particle agglomeration, affecting both flowability and processability [4–6].

Traditional methods for analyzing powder flowability involve experimental techniques such as measuring the time taken for powders to flow through a defined aperture, assessing powder density, and evaluating the angle of repose (AOR). Indices like Carr's index and the Hausner ratio provide additional insights into flow characteristics [7, 8]. Advanced instruments, such as the FT4 Powder Rheometer, can measure the torque required to rotate a blade through the powder, yielding the flow function coefficient (FFC) [9]. Jenike's flow regime classification system categorizes powders based on their flowability, identifying them as not-flowing, very cohesive, cohesive, well-flowing, or free-flowing [10]. However, these experimental approaches have notable drawbacks, particularly in terms of time and resource investment. Characterizing powder flowability can be particularly challenging when the available sample quantity is insufficient for conventional testing methods. This is especially relevant in the pharmaceutical industry, where the cost of active pharmaceutical ingredients (APIs) can be significant during the early stages of drug product development [11]; this underscores the necessity for alternative predictive methods, in particular being able to predict blend flowability based on the flow properties of individual constituents.

Recent studies have aimed to develop models that can accurately predict powder flowability by assessing various particle properties, such as size distribution, density, surface energy, surface roughness, and morphology [12–17]. However, predicting the flowability of cohesive powders remains challenging due to the intricate interplay of interparticle forces. For example, some literature [18, 19] highlighted the non-linear relationship between cohesiveness and flowability and also demonstrated that varying electrostatic charges among particles could lead to unpredictable flow behaviors [20]. Additionally, factors such as particle size and distribution can significantly influence flow characteristics, with smaller particles often exhibiting higher cohesiveness [21]. Extrinsic factors, including moisture content and processing conditions, further complicate predictions. Even minimal moisture levels could lead to agglomeration, adversely affecting flowability [22]. Pre-consolidated powders also show distinct flow characteristics compared to freshly poured powders, complicating the assessment of behavior under varying conditions. All these issues pose further challenge for blends for which ideally one would prefer to predict the blend properties based on fundamental powder properties of only the constituent APIs and excipients.

In response to these challenges, the pharmaceutical industry has increasingly turned to digital design techniques to enhance the prediction of bulk material properties and streamline early-stage development processes. Machine learning (ML) models have emerged as promising tools for predicting powder flowability. Recent advancements have demonstrated the potential of various ML techniques, including deep learning architectures like ResNet18 and Vision Transformer (ViT), to analyze microscopy images and accurately predict the flowability of cohesive drug powders at milligram scales [23]. This approach not only reduces the material requirements for testing but also minimizes the time needed for conventional flowability measurements. Techniques such as Response Surface Methodology (RSM), Partial Least Squares (PLS), and Artificial Neural Networks (ANN) have also been employed to study factors influencing fluidized bed granulation, highlighting the efficacy of ML in modeling complex relationships among multiple variables [24, 25].

Despite the advantages of ML algorithms, predicting the Flow Function Coefficient (FFC) of powders remains a challenge due to the complexity of various parameters. Previous literature [26] explored several regression models, including Linear Regression (LR) and Random Forest (RF), to predict FFC values, revealing that classification models outperformed regression approaches in accuracy. One limitation noted was that the FT4 Powder Rheometer struggles to differentiate between free-flowing powders with FFC values above 10 [27], indicating that a classification approach may provide more precise predictions of powder flowability. This raises critical questions about the optimal number of flow classifications and the most effective models for predicting flow regimes. Furthermore, this study used features of the created powder blends (*e.g.*, PSD) to predict blend flowability, as opposed to using features of the constituent components; therefore, there are also questions regarding if the properties of bulk constituents can be used to make predictions of the resulting blend.

The objective of this study was to utilize multiple ML models to predict the flow regimes of pharmaceutical components and blends across a diverse range of drug contents. Predictions were based on fundamental powder properties of only the constituent APIs and excipients, including particle size, density, surface energy, and shape. The performance of each model was rigorously evaluated against experimental data to assess accuracy and efficacy. Furthermore, the impact of dry coating on flowability was investigated, examining various coating parameters such as coating agents, surface area coverage, and the percentage of coated components in the blends. By exploring multiple flow regime scenarios, the study sought to identify the most suitable flow categories, particularly focusing on 2, 3, 4, and 5 regimes. This comprehensive approach aimed to deepen our understanding of how dry coating influenced the flow characteristics of powder blends. Ultimately, this research aimed to enhance the integration of predictive models in early-stage drug development, facilitating targeted particle engineering and informed decision-making regarding formulation and processing technologies. This could lead to significant reductions in both time and material resources, minimizing waste and optimizing the development process.

## Materials and Methods

### Materials

The study included 27 commonly used pharmaceutical powders, comprising 9 Active Pharmaceutical Ingredients (APIs) listed in Table 1, and 18 excipients listed in Table 2. These materials were selected based on their wide usage in the pharmaceutical industry. Relevant powder properties such as particle sizes (d_10_, d_50_, d_90_), Sauter mean diameter (d_3,2_), particle (true) density (ρ_p_), dispersive surface energy (γ_d_), and shape descriptors (aspect ratio, sphericity, and elongation) are presented in the tables. These properties provided a comprehensive overview of the powder characteristics and were analyzed using SHAP (SHapley Additive exPlanations) and Feature Importance to identify potential factors influencing powder flowability It is noted that most of the powders’ properties utilized in the training dataset as individual components and blends were experimentally measured by Kunnath *et al*. [27, 28]. Additionally, the study incorporated two types of pharmaceutical-grade nano-silicas (glidants) for dry coating applications to tailor the powder flowability and test the effect of nano-silica, dry coating agents. These were the hydrophobic Aerosil R972P and the hydrophilic Aerosil A200 provided by Evonik, USA. The Aerosil R972P nano-silica had a nominal particle size of 20 nm, a particle (true) density of 2650 kg/m^3^ [29], and a dispersive surface energy of 36.40 mJ/m^2^ [30]. The Aerosil A200 nano-silica had a nominal particle size of 12 nm [30], a particle (true) density of 2450 kg/m^3^ [31], and a dispersive surface energy of 42.80 mJ/m^2^ [30].

### Methods

#### Experimental Methods

##### Powder Flowability Tailoring: Dry Coating Via LabRAM

The dry coating methods, usually performed on dry particles, create high impact and shearing forces to de-agglomerate the nano-sized particles and to uniformly distribute the nanoparticles on the host surface. As the nano-size guest particles adhere to the surface of the cohesive host particles via van der Waals attractive forces [6], a nanoscale surface roughness is created, and a separation between what normally would be host-host contact occurs [32], therefore decreasing the effective interparticle attractive forces [6] and enhancing the flowability of powders without altering the particle size [33, 34]. The reduction in inter-particle adhesion is a function of the surface area coverage (SAC) by nano-size guest particles, the calculation of which has been discussed in detail elsewhere [6, 35].

**Fig. 1:**
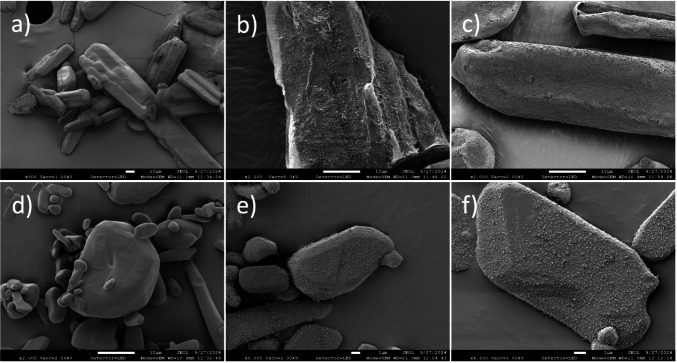
SEM images of APIs, uncoated and drycoated by hydrophobic nano-silica (R972P). For Ibuprofen 25: (**a**) uncoated, (**b**) theoretical SAC50%, (**c**) theoretical SAC100%. For micronized Acetaminophen: (**d**) uncoated, (**e**) theoretical SAC50%, and (**f**) theoretical SAC100%.

As received APIs and excipients were dry coated using a material-sparing instrument, a high-intensity vibrational mixer (LabRAM, Resodyn Corporation, USA). Approximately 50 g of either API or excipient was placed in a standard 300 mL cylindrical plastic container, acting as host particles, alongside hydrophilic or hydrophobic nano-silica as guest particles. The container was sealed with a cap before clamping into the LabRAM. The cylindrical jar filled with powder samples was then shaken for 5 min at 75 g acceleration and 60 Hz frequency, which correspond to the vibration parameters of the jar. The oscillation mixing frequency was optimized by the RAM driver control module to achieve resonance during powder mixing. The resulting images of the powder dry coating are presented in Fig. 1, comparing uncoated and dry-coated APIs (Ibuprofen 25 and micronized Acetaminophen) with varying amounts of hydrophobic nano-silica, R972P. The amount of silica used was calculated to achieve a theoretical SAC of 25%, 50%, and 100%. The weight percentage of nano-silica (Gwt. %) for the 100% SAC is calculated based on Eq. (1) [32, 35]. D_0_ and ρ_D_ represent the median particle size and true density, while d_0_ and ρ_d_ refer to the median particle size and true density of the guest particles. Examples of nano-silica dry coating applied to pharmaceutical powders with varying surface morphologies and shapes, using different amounts of nano-silica can be found in previous literature [36].

1 $$Gwt\%= \frac{SAC{d}_{0}^{3}{\rho }_{d}}{{D}_{0}^{3}{\rho }_{D}} \frac{4{D}_{0}^{2}}{{d}_{0}^{2}}\text{ x} 100\%$$

##### Particle Size Analysis

Volume-based particle size distribution of the powders is measured by the Rodos/Helos system, which is a laser diffraction particle analyzer (Rodos/M and Helos/BR, Sympatec, NJ). Size statistics in terms of d_10_, d_50_, d_90_, and d_3,2_ were measured, which are the values of the particle diameter at 10%, 50%, 90% and the Sauter mean diameter respectively in the cumulative volumetric particle size distribution. In the Rodos/Helos system, the Rodos device works by venturi principle to disperse the powder, and the HELOS unit uses laser diffraction principles of Fraunhofer Enhanced Evaluation (FREE) and Mie Extended Evaluation (MiEE) theories of light scattering to determine the particle size. PSD analyses were conducted at the dispersion pressure of 0.5 bar [27] performed 3 times and average values are reported in Tables 1 and 2.

**Table 1:** Properties of the APIs used in this Study (Particle Sizes Distribution (d_10_, d_50_, d_90_), Sauter Mean Diameters (d3,2), Particle (True) Densities (ρ_p_), Dispersive Surface Energies (γ_d_), Shape Descriptors (Aspect Ratio, Sphericity, and Elongation), and Flow Function Coefficient (FFC)

Materials	Particle Size (μm)				ρ_p_ (kg/m^3^)	γ_d_ (J/m^2^)	Aspect Ratio (-)	Sphericity (-)	Elongation (-)	FFC (-)
	*d*_*10*_	*d*_*50*_	*d*_*90*_	*d*_*3,2*_						
micronized Acetaminophen (mAPAP)	2.42	8.4	27.6	5.2	1290	0.046	0.61	0.81	0.50	1.18
Coarse Acetaminophen (cAPAP)	3.1	20.0	82.0	7.3	1290	0.041	0.55	0.81	0.45	2.66
Ibuprofen 25	8.01	28.5	58.7	12.1	1120	0.039	0.60	0.84	0.49	3.20
Ibuprofen 38	10.6	35.2	65.1	14.1	1120	0.039	0.60	0.83	0.48	4.37
Ibuprofen 50	9.5	41.0	98.0	14.4	1120	0.039	0.58	0.82	0.46	4.29
Ibuprofen 70	9.1	52.2	135.0	15.5	1120	0.039	0.59	0.83	0.47	6.70
Fenofibrate	2.5	6.9	15.0	4.5	1263	0.040	0.66	0.90	0.64	1.47
Griseofulvin	3.0	8.0	19.3	5.5	1430	0.040	0.64	0.84	0.55	2.98
Itraconazole	2.5	10.0	27.8	5.25	1365	0.036	0.63	0.83	0.22	2.06
Min	2.42	6.9	15.0	4.5	1120	0.036	0.55	0.81	0.22	1.18
Max	10.6	52.2	135.0	15.5	1430	0.046	0.66	0.90	0.64	6.70
Span	8.18	45.3	120.0	11.0	310	0.010	0.11	0.09	0.42	5.52

**Table 2:** Properties of the Excipients used in this Study (Particle Sizes Distribution (d_10_, d_50_, d_90_), Sauter Mean Diameters (d3,2), Particle (true) Densities (ρ_p_), Dispersive Surface Energies (γ_d_), Shape Descriptors (Aspect Ratio, Sphericity, and Elongation), and Flow Function Coefficient (FFC)

Materials	Particle Size (μm)				ρ_p_ (kg/m^3^)	γ_d_ (J/m^2^)	Aspect Ratio (-)	Sphericity (-)	Elongation (-)	FFC (-)
	*d*_*10*_	*d*_*50*_	*d*_*90*_	*d*_*3,2*_						
Cornstarch	8.0	14.4	22.9	15.0	1444	0.032	0.73	0.87	0.59	3.74
Granulac 200	4.8	27.9	88.5	10.3	1528	0.034	0.67	0.86	0.55	2.51
Granulac 230	3.5	21.9	56.2	7.3	1546	0.034	0.67	0.88	0.59	1.95
Lactose 120	25.3	93.9	163.0	38.0	1504	0.037	0.75	0.86	0.40	6.63
Pharmatose 350	4.5	28.2	79.6	10.1	1540	0.042	0.67	0.86	0.56	2.43
Pharmatose 450	3.65	19.2	48.6	6.2	1543	0.045	0.65	0.83	0.53	1.90
Pharmatose DCL11	48.0	115.0	202.0	85.2	1543	0.039	0.78	0.86	0.56	13.40
Sorbolac 400	1.8	8.6	20.5	4.29	1520	0.043	0.65	0.88	0.60	1.52
Avicel 101	21.2	64.2	143.0	42.9	1562	0.042	0.57	0.79	0.42	7.26
Avicel 102	31.3	117.0	165.4	49.1	1563	0.056	0.59	0.78	0.38	9.40
Avicel 105	5.6	19.3	43.4	10.8	1559	0.048	0.61	0.79	0.45	2.35
Avicel 200	53.4	185.8	322.3	100.4	1562	0.047	0.73	0.86	0.52	11.00
InhaLac 400	1.5	9.6	35.4	4.1	1642	0.044	0.66	0.90	0.64	1.81
InhaLac 500	0.88	3.2	7.9	2.11	1739	0.043	0.60	0.91	0.71	1.54
Lactochem Microfine	1.3	4.2	22.0	3.23	1734	0.043	0.62	0.85	0.59	1.40
Lactochem Regular	65.0	45.0	103.0	11.8	1734	0.043	0.66	0.86	0.53	3.36
Lactohale 220	2.9	12.5	32.1	6.4	1780	0.044	0.65	0.84	0.54	1.60
Lactohale 230	1.9	8.5	25.5	4.5	1710	0.043	0.64	0.86	0.58	1.35
Min	0.88	3.2	7.9	2.11	1444	0.032	0.60	0.91	0.38	1.35
Max	65.0	185.8	322.3	100.4	1780	0.056	0.75	0.78	0.71	13.40
Span	64.1	182.6	314.4	98.29	336	0.024	0.15	0.13	0.33	12.05

##### Particle (True) Density Analysis

The true density of the APIs and excipients were experimentally measured using a pycnometer (NOVA 3200, Quantachrome Instruments, Boynton Beach, FL, USA) with helium gas. Each powder was measured five times. Table 1 and 2 show their average values.

##### Surface Energy Analysis

A particle’s total surface energy was evaluated using automated inverse gas chromatography (SEA-IGC; Surface Energy Measurement Systems Ltd., UK). The results of the sum of the Liftshitz-van der Waals dispersive surface energy and the polarity attained via the Schultz method [37] are shown in Tables 1 and 2. A more complete description of the sample preparation and surface energy analysis employed in this study may be found elsewhere [27, 30].

##### Particle Morphology Analysis: Scanning Electron Microscopy

A Field Emission Scanning Electron Microscope (SEM, EM JSM- 7900 F, JEOUL Ltd., Peabody, MA, USA) was used to examine particle surface morphology. Powder samples have been attached with a double-sided carbon type and placed on an aluminum stub and sputter-coated with Carbon (Q150 T, Quorum Technologies Ltd., Laughton, East Sussex, England) to improve conductivity prior to SEM imaging [36].

##### Particle Shape Analysis: Dynamic Imaging-Based

All materials were analyzed for particle shape using a QicPic/Rodos (Sympatec, USA). This device analyzes solitary particles utilizing high-speed dynamic image analysis [38, 39], distributing powders into primary particles (Rodos) and capturing 2D pictures in real-time (QicPic) before transforming the image data into shape and PSD data. Triplicate measurements were taken at a dispersion pressure of 0.5 bar to ensure proper particle dispersion. For the purpose of this study, the aspect ratio (ratio of the minimum to maximum FERET diameter [Sympatec, USA]), sphericity (ratio of the perimeter of the equivalent circle, P_EQPC_, to the real perimeter, P_real_. [Sympatec, USA]), and elongation (ratio of diameter and length) are assessed. Details of sample preparation and measurement process can be found in previous literature [27].

##### Powder Bulk Properties: Flow and Bulk Density Assessments

The bulk powder flowability and bulk density were measured using an FT4 powder rheometer (FT4, Freeman Technology, UK). This was done for both the individual powder components as well as the blends, with and without dry coating with nano-silica. All the powder samples were tested in triplicate and average values are reported in Tables 1 and 2. Detailed experimental procedure is described elsewhere [34, 40]. The conditioned bulk density was measured by placing the sample in a 25 mm × 25 mL glass-splitting cylindrical vessel. After the conditioning step, any excess powder was removed, and the mass of the 25 mL sample was recorded. The bulk density was then calculated based on the recorded mass and the known volume. For the shear cell test, the sample is placed in a 25 mm × 10 mL glass-splitting cylindrical vessel. After the conditioning test using a 23.5 mm conditioning blade, a vented piston is used to compress the powder bed to 3 kPa. This piston is then replaced by a shear head, which measures the shear stress at different normal stresses. From these measurements, the flow function coefficient (FFC), defined as the ratio of consolidation stress (σ_1_) to the unconfined yield stress (σ_c_) was obtained. Schulze [41] defined a classification of powder flow behavior, similar to that of Jenike [10], according to the flow function coefficient (FFC). The larger the FFC, the better the bulk solid flow: i.e., FFC < 1, not flowing; 1 < FFC < 2, very cohesive; 2 < FFC < 4, cohesive; 4 < FFC < 10, well-flowing; and FFC > 10, free-flowing. For direct compression downstream processing, a conditioned bulk density greater than 0.38 g/ml and an FFC greater than 6.8 are desirable [42].

In this study, as the purpose is to test the performance of the machine learning models in predicting the FFC classification of both individual components and blends, the FFC classification proposed by Schulze [41] was rearranged into 2, 3, 4, and 5 flow regimes, as shown in Table 3.

**Table 3:** Flow classifications for Numerical Flow Function Coefficient (FFC) Values, Rearranged from Schulze [41]

2 Flow Regimes		3 Flow Regimes	
FFC Value	Flow Classification	FFC Value	Flow Classification
FFC < 6	Not-Well-Flowing	FFC < 4	Cohesive
FFC ≥ 6	Well-Flowing	4 ≤ FFC < 10	Well-Flowing
		FFC ≥ 10	Free-Flowing
4 Flow Regimes		5 Flow Regimes	
FFC Value	Flow Classification	FFC Value	Flow Classification
FFC < 2	Very Cohesive	FFC < 2	Very Cohesive
2 ≤ FFC < 4	Cohesive	2 ≤ FFC < 4	Cohesive
4 ≤ FFC < 10	Well-Flowing	4 ≤ FFC < 6	Semi-Cohesive
FFC ≥ 10	Free-Flowing	6 ≤ FFC < 10	Well-Flowing
		FFC ≥ 10	Free-Flowing

##### Binary Blends Preparation

The blending parameters were held constant across all formulations to solely focus on evaluating the powder properties. These fixed parameters included a filling level of 50 g, a mixing time of 12 min, and a mixing intensity of 25 rpm in a 0.5-pint V-shaped blender. The variables that were adjusted included the percentage of API loading, the amount of silica (%SAC), and the types of silica used, depending on the training and testing purposes.

##### Machine Learning Methods

To evaluate predictive performance across different methodologies, multiple machine learning (ML) models were employed. Both unsupervised and supervised ML techniques were applied, including Principal Component Analysis (PCA) and K-means clustering for unsupervised learning, as well as six supervised learning algorithms: Random Forest (RF), Extreme Gradient Boosting (XGBoost), Support Vector Machine (SVM), Logistic Regression (LR), K-Nearest Neighbor (KNN), and Multi-layer Perceptron (MLP). The primary objective was to assess the models'effectiveness in predicting powder flow regimes for individual components and blends, based on various particle properties including size, density, surface energy, and shape, while also examining the impact of powder dry coating. To ensure that the input features were on a comparable scale and to prevent features with larger numerical scales from disproportionately influencing the models, the data were standardized using StandardScaler. This process centered the data around a mean of zero and scaled the variance to 1, thereby normalizing all input features prior to model training. Hyperparameter tuning was conducted using GridSearchCV to optimize the performance of each model by selecting the best possible parameter settings. This process involved tuning parameters such as max_depth (for Decision Trees), n_estimators (for Random Forests), n_neighbors (for K-Nearest Neighbors), and the kernel type (for Support Vector Machines), ensuring optimal configurations for improved accuracy. The analysis was conducted using the Anaconda Spyder (Scientific Python Development Environment) platform. The ML models were implemented using Scikit-Learn 1.3.0 with Python 3.11.5 as the programming language. Pandas 2.1.0 and NumPy 1.25.2 were employed for processing tabular data, while Matplotlib 3.7.2 and Seaborn 0.12.2 were used for visualizing both the dataset and modeling outcomes.

###### Dataset Construction

The training dataset comprised 9 active pharmaceutical ingredients (APIs) and 18 excipients. In total, there were 89 single components and 321 original blends that were used to train the predictive models. The features of the blends included all the aforementioned constituent API and excipient properties (PSD, surface energy, density, shape, etc*.*), percent API and percent excipient in the blend, a categorical feature indicating if the API and/or excipient was coated and the type of silica used, and surface area coverage of silica. To include individual components in the dataset, each individual API and excipient was included by “mixing” it with a corresponding API or excipient with all features set to zero and comprising 0% of the blend. This approach was necessary to enable the individual powders and blends to be treated within the same dataset, allowing for the inclusion of powders containing only a single component. In addition, to investigate the distinct characteristics of single components, separate training, and analysis were conducted for them alone. The details of the blend formulations are presented in Table 4, which outlines the various combinations of Active Pharmaceutical Ingredients (APIs), excipients, drug loading percentage, %SAC (surface area coverage), and silica type used in the blend preparations. It is important to note that not all combinations of these parameters were applied to every API and excipient. The combinations outlined in Table 4 yielded a total of 410 blends for testing.

**Table 4:** Details of the Binary Component Blend Formulations of the Training Dataset

APIs	Excipients	Drug Loading	%SAC	Silica Type
cAPAP	Avicel 101	25%	0%	A200
mAPAP	Avicel 102	50%	25%	R972P
Fenofibrate	Avicel 105	75%	50%	
Griseofulvin	Avicel 200		100%	
Ibuprofen 25	Cornstarch			
Ibuprofen 38	Granulac 200			
Ibuprofen 50	Granulac 230			
Ibuprofen 70	InhaLac 400			
Itraconazole	InhaLac 500			
Dummy"z"	Lactohale 220			
	Lactohale 230			
	Lactose 120			
	Lectochem Microfine			
	Lectochem Regular			
	Pharmatose 350			
	Pharmatose 450			
	Pharmatose DCL11			
	Sorbolac 400			
	Dummy"a"			

###### Model Validation

(I) To validate the performance of the ML models, new blend formulations were experimentally prepared, using the API and excipients that were included in the original dataset but the API loading percentages were varied in proportions not included in the original dataset. The mAPAP and Avicel 102 were selected as the testing API and excipient, respectively. They were blended with API loading of 5%, 35%, 65%, and 95%. Additionally, the impact of nano-silica dry coating and the type of nano-silica were also evaluated. The mAPAP was coated with either hydrophilic (A200) or hydrophobic (R972P) nano-silica at 50% SAC and blends were also prepared without any coating (0% SAC). The FFCs of these test blends were then measured and compared to the ML model predictions for 2, 3, 4, and 5 flow regimes.

(II) For a comprehensive validation of the ML model predictions, the models were further tested on their ability to predict the behavior of an API that was not presented in the original training dataset. Specifically, the 59 data points that included Ibuprofen 25 (which accounted for 14% of the 410-sample dataset) were removed from the training data. After the ML models were trained on the reduced dataset, the Ibuprofen 25-containing data was reintroduced and used to test the models'performance. The predicted flow regimes (2, 3, 4, and 5 classifications) for these Ibuprofen 25 blends were then compared to the experimentally measured values. This allowed for a rigorous evaluation of the ML models'capability to accurately predict the behavior of an unknown API that was not initially included in the training process.

###### Unsupervised Learning Algorithms

Two unsupervised learning algorithms, Principal Components Analysis (PCA) and K-means clustering were employed to analyze individual components and blends within the dataset. PCA was applied as a data preprocessing step to reduce the dimensionality of the dataset, facilitating easier visualization. K-means clustering was then performed to identify the optimal number of clusters, utilizing the Within-Cluster Sum of Squares (WCSS) and the elbow method. WCSS evaluates the total variance within each cluster, providing insight into the effectiveness of the clustering algorithm in grouping the data points. The performance of the K-means algorithm was assessed by calculating the number of misclassifications relative to the true flow classifications, providing a comparative measure of clustering accuracy.

###### Classification Models

Classification models were developed using a 70:30 split of the dataset, these models were then used to predict powder flow phenomena in the test set. To evaluate the performance of each classification model, a Leave-One-Out Cross-Validation (LOO-CV) approach was employed. The average LOO-CV values were calculated and compared across the 2, 3, 4, and 5 flow regimes defined in Table 3. Leave-One-Out cross-validation (LOO-CV), a robust and unbiased method for evaluating the models'performance, was performed to ensure generalization and to avoid the fact that the mean squared error (MSE) can vary significantly depending on the specific observations used in the training and testing sets [43, 44]. Feature Importance and SHAP analysis were used to assess the contribution of each variable to the model’s predictions. A higher score value indicates a more significant contribution. Additionally, a confusion matrix was generated to evaluate the accuracy and misclassification rates of the RF model.

## Results and Discussion

### Particle Size and Morphology

**Fig. 2:**
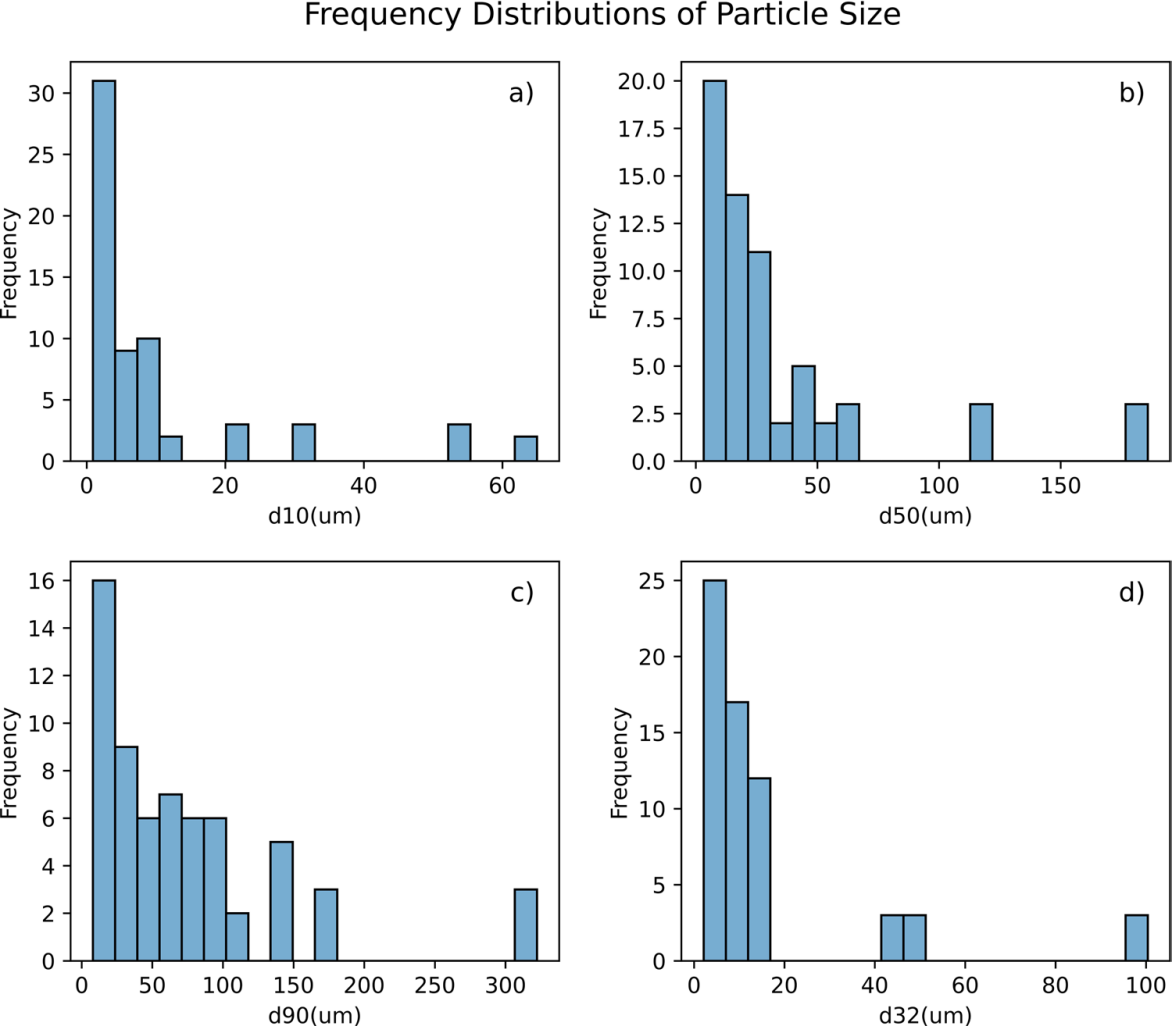
Frequency of particle size distribution values of powders used in this study: (**a**) d10, (**b**) d50, (**c**) d90, and (**d**) sauter mean diameters (d3,2).

The particle size analysis of the Active Pharmaceutical Ingredients (APIs) and excipients used in this study was conducted using Rodos/Helos PSD measurements, with a focus on four key particle size descriptors: d_10_, d_50_, d_90_, and the Sauter Mean Diameter (d_3,2_). Table 5 presents the range and median values of these descriptors. The analysis revealed a wide variety of particle sizes among the APIs and excipients, highlighting considerable differences in the distribution of both fine and coarse particles. Frequency distributions for these particle size descriptors are illustrated in Fig. 2a-d. The plots indicate that the majority of particles for d_10_, d_50_, d_90_, and d_3,2_ are skewed towards the lower end of the size spectrum, reflecting a dominance of finer particles in the sample. The median sizes observed across the different descriptors suggest that these materials primarily consist of fine particles, which may influence powder flowability in pharmaceutical formulations.

**Table 5:** Range and Median of Particle Size Characteristics for APIs and Excipients used in this Study

Particle Size Characteristics	Particle Size Range (µm)	Particle Size Median (µm)
d_10_	0.88–65.0	4.5
d_50_	3.2–185.8	20.0
d_90_	7.9–322.3	56.2
Sauter Mean Diameter (d_3,2_)	2.11–100.4	10.1

**Fig. 3:**
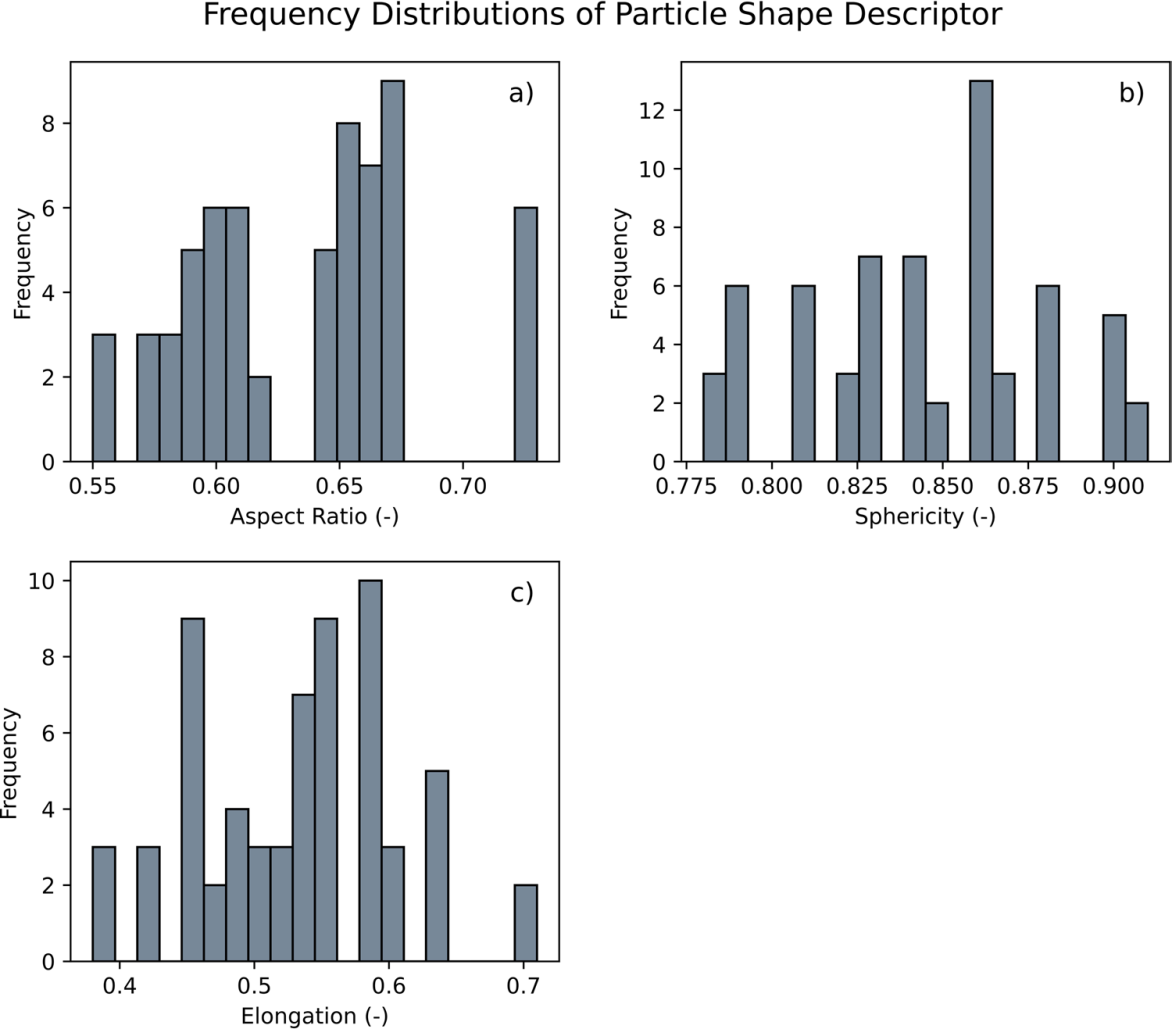
Frequency of particle shape values of powders used in this study: (**a**) aspect ratio, (**b**) sphericity, and (**c**) elongation.

Table 6 provides the range and median values of key particle shape descriptors, including aspect ratio, sphericity, and elongation assessed using the Sympatec Gradis/QicPic. The median aspect ratio of 0.64 indicates a tendency towards an elongated shape, while still falling within a moderate range. With a median sphericity of 0.84, the particles can be characterized as relatively spherical. Notably, there is considerable variation in elongation, with a median value of 0.53. Histograms depicting the frequency distributions for each particle shape descriptor are shown in Fig. 3. The distribution of aspect ratio (Fig. 3a) shows a peak around 0.64, aligning with the median value, and the presence of multiple peaks suggests a diversity in particle shapes. The sphericity distribution (Fig. 3b) appears more uniform, indicating that most particles closely resemble spherical shapes. In contrast, the elongation distribution displays a broad range with multiple peaks, reflecting the variety of particle shapes present in the sample.

**Table 6:** Range and Median of Particle Shape Descriptors for APIs and Excipients Used in this Study

Particle Shape Descriptors	Particle Shape Range (-)	Particle Shape Median (-)
Aspect Ratio	0.55–0.73	0.64
Sphericity	0.78–0.91	0.84
Elongation	0.38–0.71	0.53

### Powder Flowability and Flow Classification Prediction

#### Unsupervised Algorithms

Using PCA for dimensionality reduction not only simplifies the data but also helps in enhancing the clarity of the clusters, making it easier to identify and interpret meaningful patterns. This aligns with the objectives of K-means as effectively facilitating clustering through its dimension reduction capabilities [45]. Both individual and blend datasets were analyzed to identify the Principal Components (PCs) along with their eigenvalues and eigenvectors. The analysis revealed that the top two principal components explain 95.4% of the variance in the individual component dataset and 97.9% in the blend dataset. This demonstrates the effectiveness of these principal components in capturing the underlying structure of the data. Hence, these components were selected for K-means clustering.

To identify the optimal number of clusters (k) for individual components and blends, the unsupervised K-means clustering algorithm was employed, utilizing the Within-Cluster Sum of Squares (WCSS) and the elbow method. The analysis revealed that the optimal number of clusters was 4 for individual components and 3 for blends. The flowability of both datasets was then classified according to their FFC values, aligning with the optimal k determined from the analysis and flow regimes in Table 3. The distribution of observations across the different flow regimes is presented in Table 7.

**Table 7:** The Distribution of Observations Across the Different Flow Regimes of Individual Components and Blends

Individual Components (k = 4)			Blends (k = 3)		
FFC Values	Flow Classification	Number of Observations	FFC Values	Flow Classification	Number of Observations
FFC < 2	Very Cohesive	10	FFC < 4	Cohesive	147
2 ≤ FFC < 4	Cohesive	14	4 ≤ FFC < 10	Well-Flowing	179
4 ≤ FFC < 10	Well-Flowing	21	FFC ≥ 10	Free-Flowing	84
FFC ≥ 10	Free-Flowing	18			

**Fig. 4:**
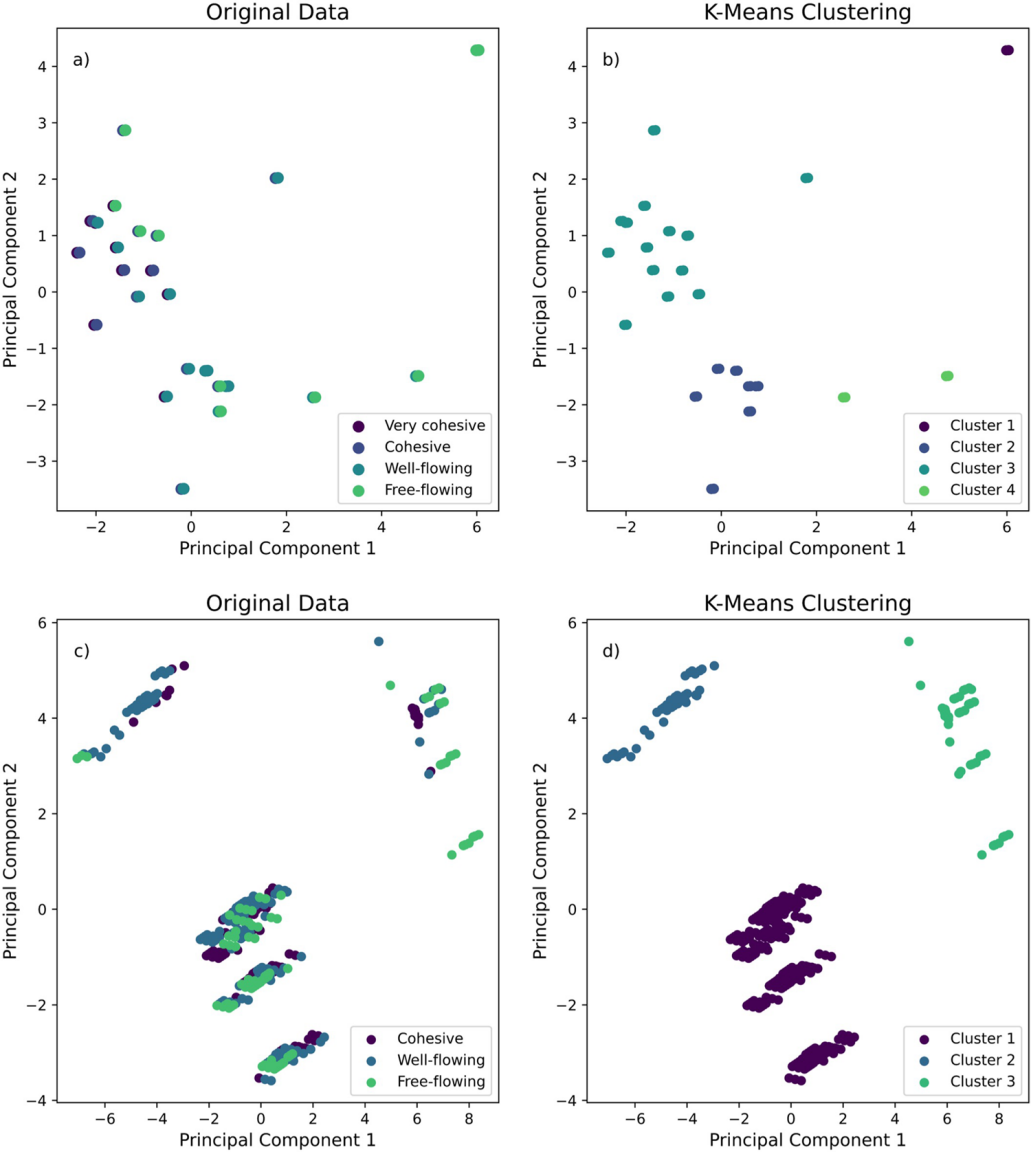
K-means clustering (at optimal *k*) and principal components analysis of individual components (**a**-**b**) and blends (**c**-**d**) datasets.

**Fig. 5:**
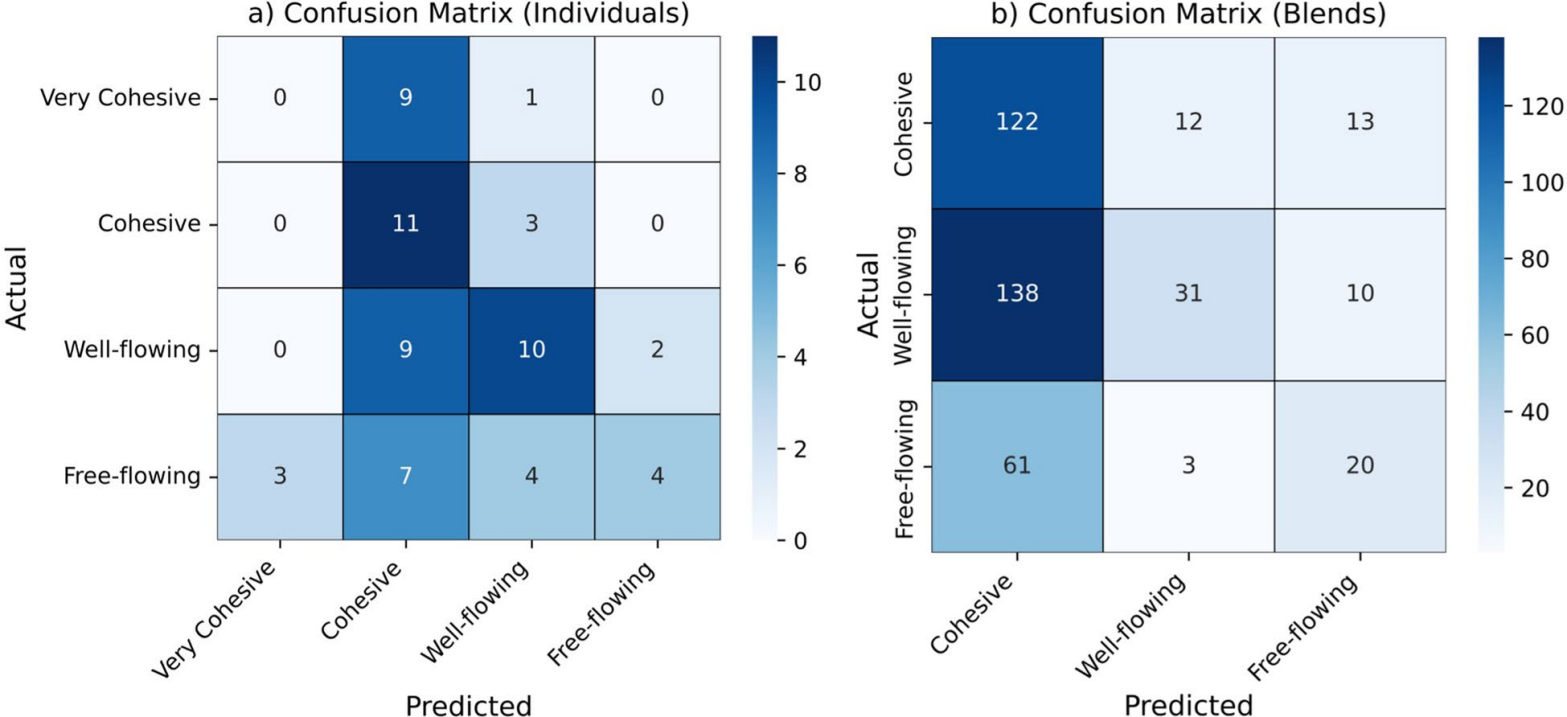
Confusion matrices for K-means clustering: powder flow classification predictions for individual components (**a**) and blends (**b**) datasets.

The separation of clusters identified by K-means clustering using the optimal number of clusters (k) is illustrated in biplots presented in Fig. 4b, d. These visualizations reveal distinct groupings, highlighting the differences in flow classification results when compared to the original flow classifications, which are plotted side by side (Fig. 4a, c). The predictive performance of the model, measured as the proportion of correct predictions relative to the total number of instances, yielded accuracies of 40% for the individual dataset and 42% for the blends. Additionally, the confusion matrices for each flow classification are displayed in Fig. 5 for both datasets, providing a detailed breakdown of prediction outcomes.

The results indicate that unsupervised algorithms, such as PCA and K-means, may not be suitable for predicting pharmaceutical flow regimes, as they did not correlate well with the observed powder flow behaviors. This suggests that these methods may lack the necessary sensitivity to capture the complexities intrinsic in pharmaceutical powders. Therefore, further investigation utilizing supervised algorithms is necessary to enhance prediction accuracy and better understand the relationships between the powder properties and flow regimes.

#### Supervised Algorithms

Six ML classification models (RF, XGBoost, SVM, LR, KNN, and MLP) were applied to predict the flow regimes of individual components and blends. The accuracy of each model was compared across 2, 3, 4, and 5 flow regimes, based on their FFC values as outlined in Table 3. Bulk powder properties, including particle sizes, density, surface energy, and shape data were utilized as input features for the models, as described in the Methods section.

**Fig. 6:**
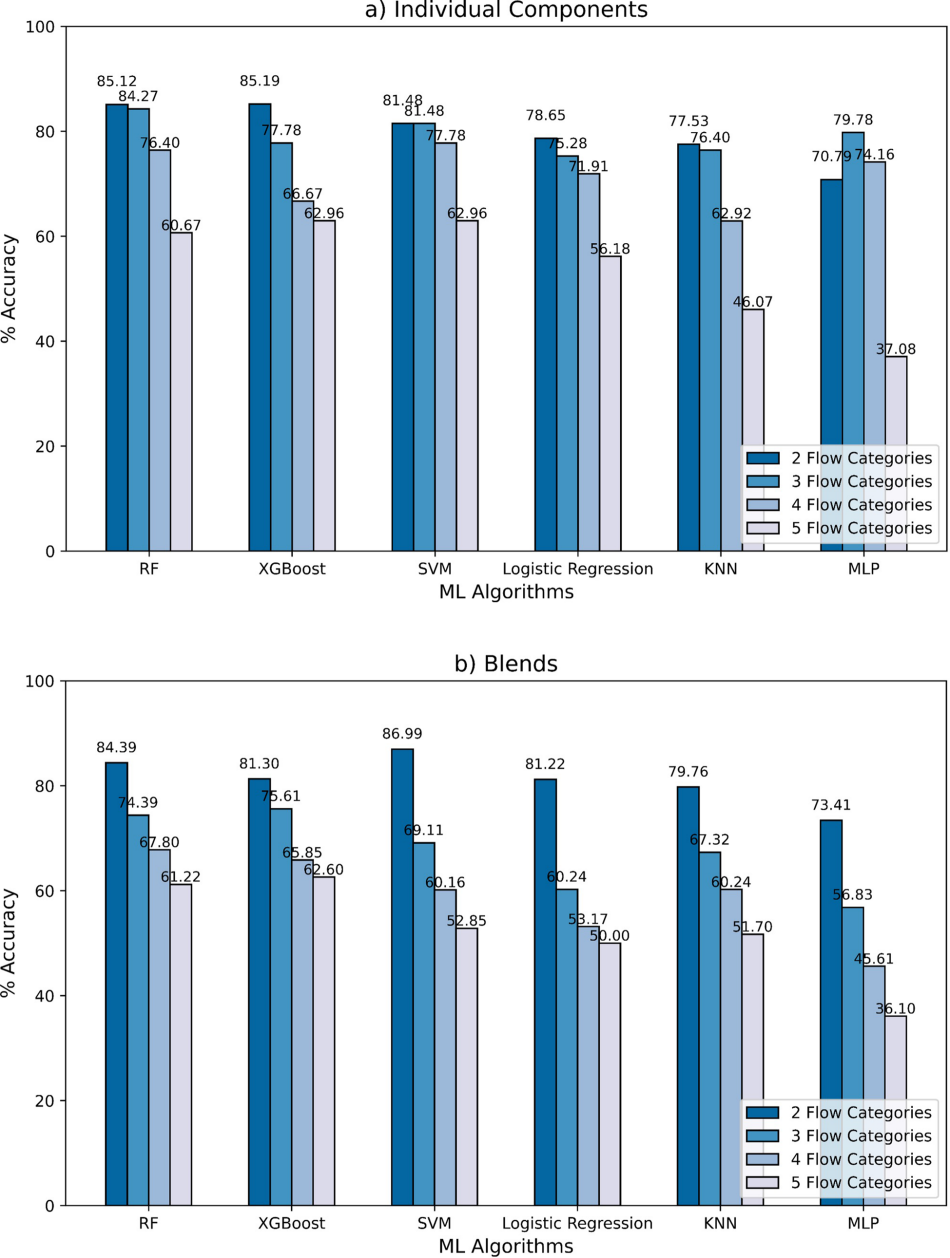
The model performance comparison across all powder flow regimes of the (**a**) individual components and (**b**) blends datasets.

Figures 6a and b present a summary of the model performance comparison across all the powder flow regimes for the individual components and blends datasets, respectively. Across both datasets, all the ML models demonstrated strong performance when the data was more granular, specifically with only two powder flow regimes, achieving a maximum evaluation accuracy of 85% and 87% for individual components and blends, respectively. However, as the number of powder flow categories increased, the accuracy declined, indicating a relationship between the complexity of the classification task and model performance. This trend underscores the challenges associated with accurately predicting flow regimes as the number of categories rises.

In the individual components dataset, the RF and XGBoost models achieved the highest accuracy when classified into two flow categories, reaching 85.12% and 85.19%, respectively. The performance of all models declined as the number of flow categories increased. Upon increasing the number of flowability categories to 3, all 6 of the models retained an average accuracy of approximately 79%, with RF performing the best at 84.27%. However, only 4 of the models (RF, SVM, LR, and MLP) retained above 70% accuracy for 4 flowability categories. Finally, at the most granular 5 flowability categories, the best-performing models were XGBoost and SVM at approximately 63%, with RF closely following at 60.67% accuracy. The lowest accuracy was observed for the MLP at 37.08% for five flow categories; the KNN model also exhibited notably lower accuracy, particularly in the more complex classifications.

Similarly, in the blends dataset, the models all showed relatively the same performance as with the individual powders, with all models performing the best with 2 flow regime categories, and accuracy decreasing as the number of regimes increased. RF and XGBoost still performed very well, at 84.39% and 81.30%, respectively, in 2 flow regimes; interestingly, however, the SVM performed the best at 2 flow regimes with an accuracy of 86.99%. However, at all other numbers of flow regime categories, RF and XGBoost eventually performed the best, even at 5 flow regimes with accuracies of approximately 61%. The trend across both datasets indicates that as the number of flow categories increases, the accuracy of all models tends to decrease, highlighting the challenges of accurately classifying more complex flow regimes.

Overall, RF and XGBoost consistently outperformed other algorithms across both datasets, demonstrating their robustness in handling the classification tasks, while MLP and KNN showed significant drops in accuracy as the classification complexity increased.

##### Random Forest

The comparison of model performance highlights the crucial of selecting suitable ML models that align with the characteristics of the data and the specific classification objectives. To enhance the prediction of powder flow classifications and conduct a more in-depth analysis of Feature Importance, the RF algorithm has been chosen for further investigation as it displayed good performance across both individual powders and blends across all numbers of flowability categories.

###### RF Powder Flow Classification Prediction

**Fig. 7:**
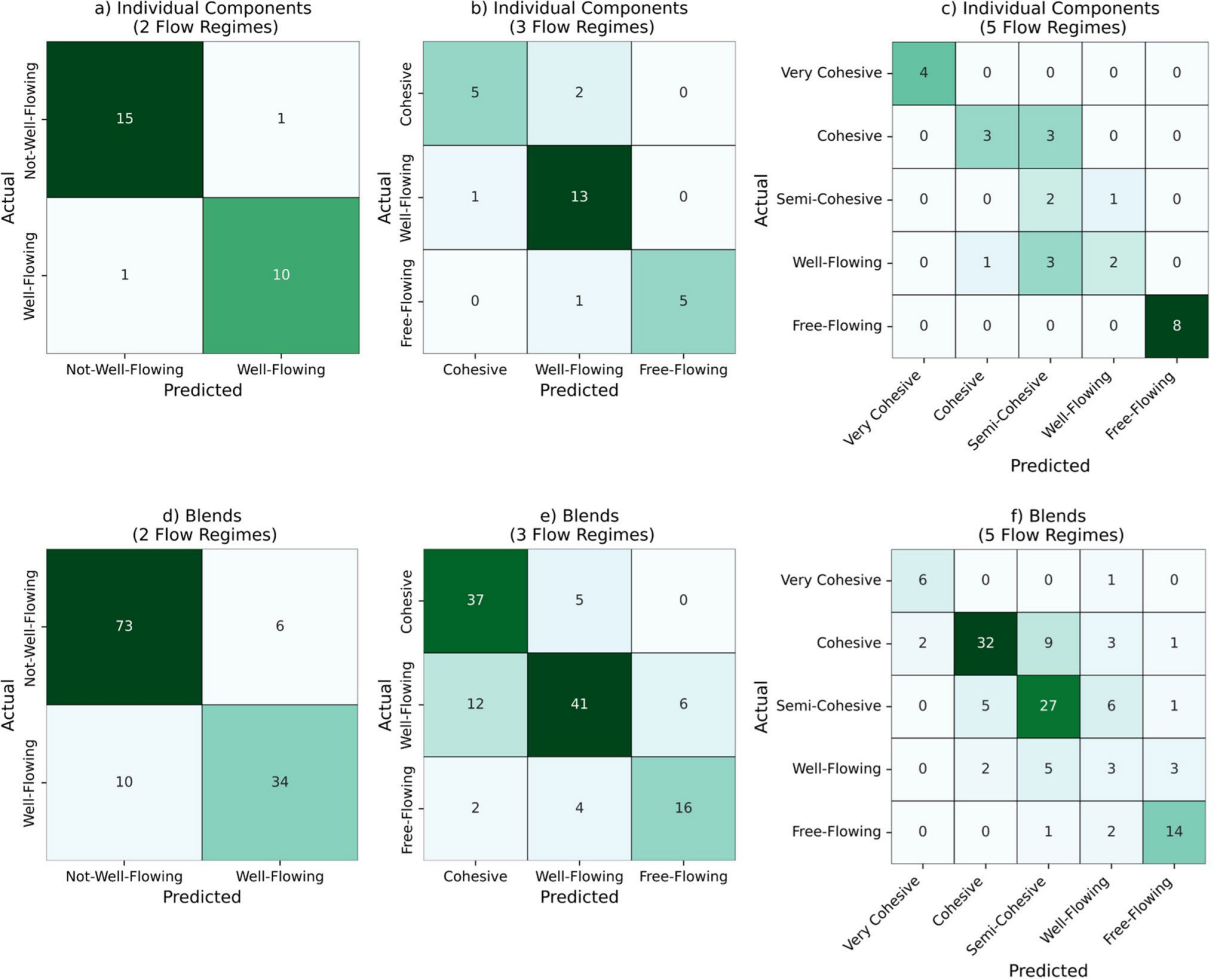
Confusion matrices for the random forest model: individual components (**a**-**c**) and blends (**d**-**f**) datasets across 2, 3, and 5 powder flow regimes.

The confusion matrices for the RF model, presented in Fig. 7a-f, provide insights into the model's performance across both individual components and blends datasets for varying powder flow regimes.

In the individual components dataset (Figs. 7a-c), the RF model demonstrates solid predictive capability, particularly with two flow regimes (Fig. 7a), achieving 15 correct predictions for"Not-Well-Flowing"and 10 for"Well-Flowing."However, it misclassified 1 instance of"Well-Flowing"as"Not-Well-Flowing."As the number of flow regimes increases to three (Fig. 7b), the model maintains solid performance, correctly predicting the majority of categories but experiencing a slight increase in misclassifications. For example, it incorrectly classified one"Cohesive"sample as"Well-Flowing."The confusion matrix indicates a more complicated performance in the five flow regimes scenario (Fig. 7c). The model correctly identified 4"Very Cohesive"samples but struggled with misclassifying several instances among the"Cohesive,""Semi-Cohesive,"and"Well-Flowing"categories. While this demonstrates that as the granularity of flowability categories increases, the model has difficulty in differentiating between these “middle” values, it did correctly predict all"Free-Flowing"samples, highlighting its effectiveness in this classification.

Turning to the blends dataset, the RF model shows even higher accuracy in the two flow regimes (Fig. 7d), with 73 correct predictions for"Not-Well-Flowing."The model's performance in this case reflects its robustness, with only 6 misclassifications. In the three flow regimes (Fig. 7e), the model continues to perform well, though some misclassifications are evident, particularly in distinguishing between"Cohesive"and"Well-Flowing,"with 17 total misclassifications between these two categories. The model correctly identified 41"Well-Flowing"samples but misclassified some as"Cohesive."Meanwhile, there were only 6 misclassified “Free-Flowing” powders. Finally, in the five flow regimes (Fig. 7f), the confusion matrix reveals a complex pattern of predictions, with the model excelling in identifying"Cohesive"samples (32 correct predictions) while encountering difficulties with"Semi-Cohesive"and “Well-Flowing” classifications. Once again this demonstrates that these machine learning models seem to be able to well-distinguish powder flowability at the extremes (very cohesive or very free flowing), but as the granularity increases, the models have more difficulty in distinguishing the moderate flowability values.

Overall, the performance comparison presented in Table 8 further corroborates these results. The RF model exhibits strong accuracy across all datasets, with the highest accuracy of 0.85 for two flow regimes in the individual components dataset and 0.86 for the blends dataset. However, the classification accuracy decreased as the number of flow categories increased, with the lowest accuracy observed at 0.74 for five regimes in the individual components dataset and 0.63 in the blends dataset.

**Table 8:** Performance Comparison of the Random Forest Model Across 2, 3, and 5 Flow Regimes

Individual Components				Blends			
Flow Classifications	Accuracy	ROC-AUC Score	F1-score	Flow Classifications	Accuracy	ROC-AUC Score	F1-score
2 Regimes	0.85	0.85	0.84	2 Regimes	0.86	0.85	0.86
3 Regimes	0.81	0.97	0.82	3 Regimes	0.76	0.89	0.76
5 Regimes	0.74	0.84	0.80	5 Regimes	0.64	0.84	0.63

###### The Impact of Powder Dry Coating

**Fig. 8:**
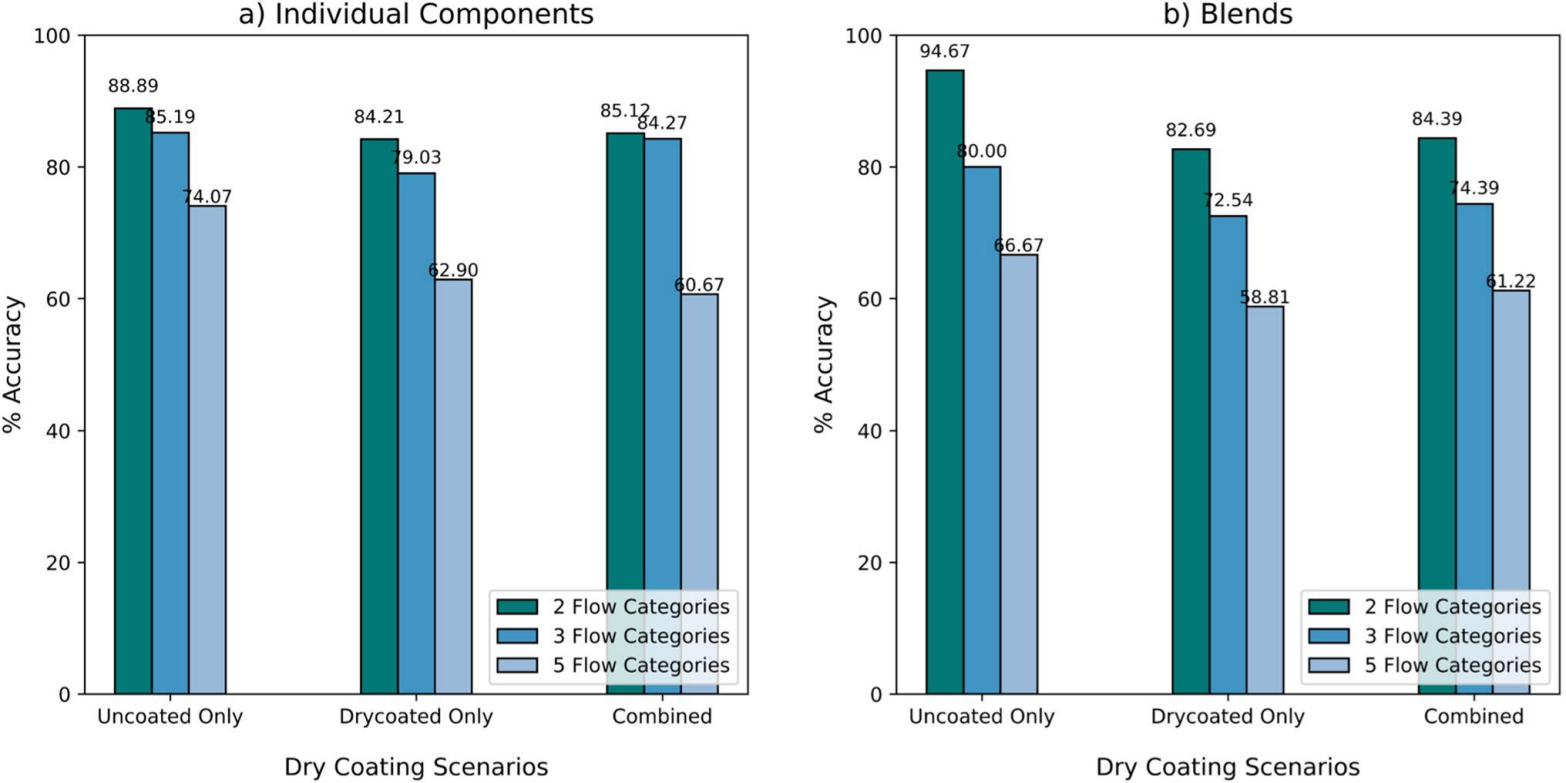
Comparison of model performance across powder flow regimes by powder dry coating scenario: uncoated-only, dry-coated-only, and combined, for individual components (**a**) and blends (**b**).

To evaluate the impact of dry coating on model performance, classification accuracy was assessed separately for uncoated and dry-coated powders. The results indicated that the model achieved its highest accuracy with uncoated powders, reaching 88.89% for individual components (Fig. 8a) and 94.67% for blends (Fig. 8b) when predicting two flow regimes. However, the introduction of dry-coated powders led to a decline in accuracy, likely due to modifications in powder properties that increased the complexity of model predictions.

Despite this decline, the overall trend remained consistent. Models performed better when the classification task was less granular. Specifically, as the number of flow regimes increased from two to five, accuracy decreased across both uncoated and dry-coated powders. This trend highlighted classification complexity as a key factor affecting predictive performance, with dry coating further amplifying this challenge.

###### Feature Importance and SHAP Analysis

**Fig. 9:**
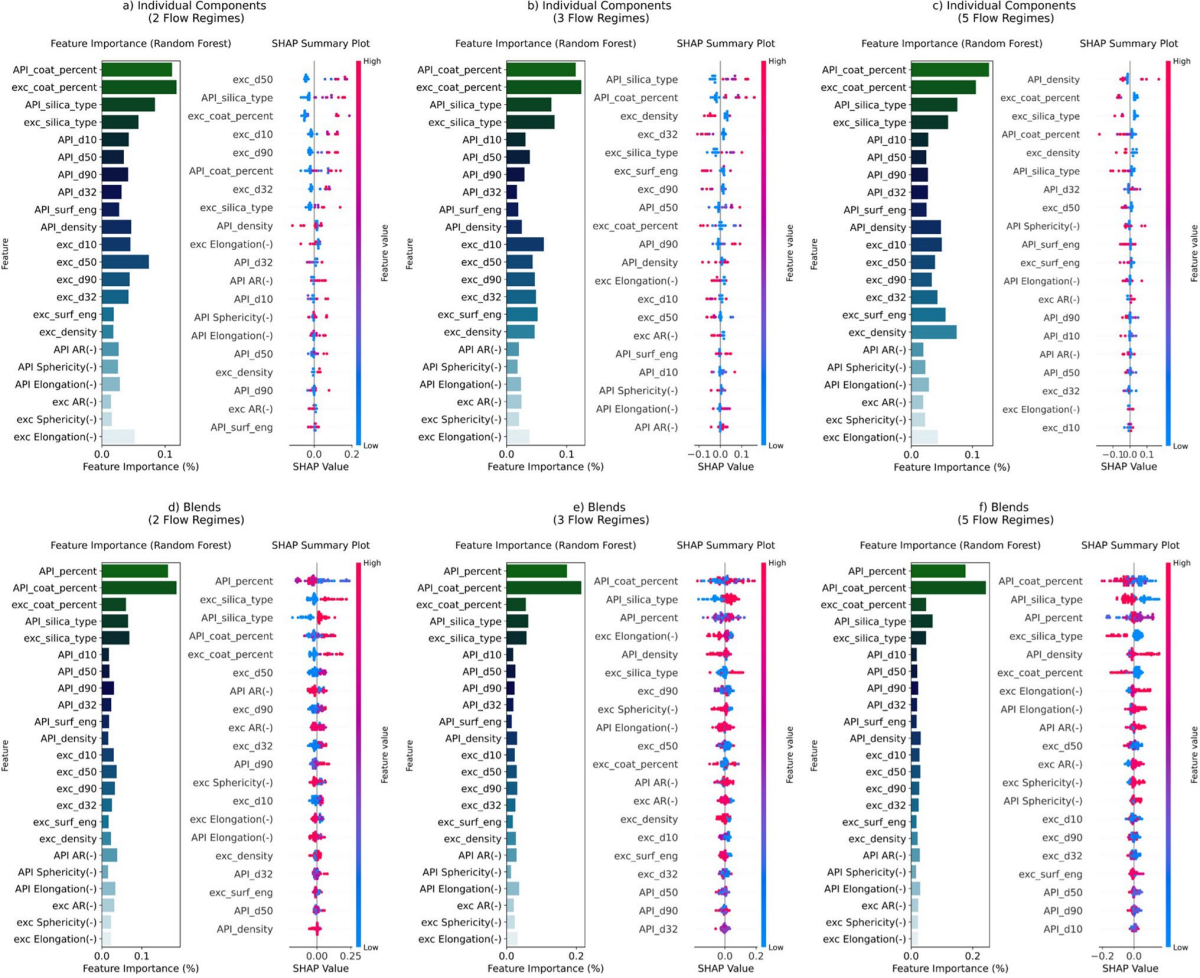
Feature importance analysis and SHAP (SHapley Additive exPlanations) for the random forest model: individual components (**a**-**c**) and blends (**d**-**f**) datasets across 2, 3, and 5 powder flow regimes.

The Feature Importance analysis for the RF model in powder flow classification, illustrated in Figs. 9a-f, highlighted the key attributes that the model relies on to differentiate between various powder flow regimes across both individual components and blends datasets.

In the individual components dataset (Figs. 9a-c), coating parameters (API_coat_percent and exc_coat_percent) emerged as the most important features influencing powder flow regime classification. This highlighted the significant role of nano-silica dry coating in improving powder flowability, likely due to its ability to modify surface interactions and reduce the cohesion forces without changing particle sizes. Bulk parameters, including particle size (e.g., d_10_, d_50_, d_90_, and d_3,2_ of both API and excipient), and density (API_density and exc_density), followed behind the coating parameters, reflecting their strong contribution to model performance. SHAP analysis further confirmed this trend, indicating that coating parameters and particle size features consistently had the highest SHAP values across different flow regimes. In Fig. 9, the SHAP features were sorted by order of importance, which highlighted the relative contribution of each feature to the model’s performance. This sorting provided a visualization of the most influential parameters in predicting powder flow regimes. The particle size distribution of excipients exhibited greater significance compared to that of APIs. Notably, the surface energy (API_surf_energy and exc_surf_energy) of the powders showed moderate importance. Among all the shape descriptors, elongation (API elongation and exc elongation) appeared to be the most significant, outweighing both aspect ratio and sphericity.

In the blends dataset (Figs. 9d-f), the API coating percentages were the most important predictors across all flow regimes, reinforcing the critical role of surface modification in flowability prediction when multiple components were mixed. The second most important feature was the API percentage, as the ratio of API to excipients influences particle interactions and adjusts the blends’ particle size distribution and surface characteristics. A higher concentration of excipients can improve flowability by reducing inter-particle forces, making this balance crucial for achieving optimal flow properties in the pharmaceutical manufacturing process. Interestingly, the excipient coating percentage was less influential than the API, a similar trend also observed with the types of nano-silica used. Unlike the individual component dataset, SHAP analysis revealed that particle size distribution had a lower impact on flow classification predictions in the blends dataset, supporting the findings from feature importance rankings. The importance of API surface energy was comparable to that of shape descriptors like sphericity, though aspect ratio and elongation were found to be more influential, as reflected by their higher SHAP values. While previous literature suggested that powder flowability is more strongly dependent on size and shape than on surface energy [26], this general trend requires careful interpretation. As the number of flow regimes increases, the significance of size and shape features appears to diminish, while the importance of surface energy and density increases, suggesting their changing relevance in more complex classifications.

In conclusion, both the individual components and blends datasets revealed that coating parameters played a crucial role in predicting powder flow regimes, underscoring the importance of surface modifications. Bulk properties such as particle size and density were also significant contributors, with elongation emerging as the most influential shape descriptor in individual components and aspect ratio in blends. Interestingly, in the blends dataset, excipient particle size distribution was more important than that of APIs, while the importance of size and shape features diminished as flow complexity increased. These findings highlight the dynamic interplay of physical properties and surface characteristics in pharmaceutical powder flowability, especially when multiple components are involved.

### Model Validation

#### Unknown API Percentage

As described in Section"Model Validation", experiments were conducted to evaluate the performance of the RF model when predicting powder flow regimes for unknown API percentages not included in the training dataset. The predicted regimes were compared against the actual flow regimes, and the results are presented in Table 9.. The table highlights areas of misclassification, with the shaded cells indicating instances where the RF model's predicted flow regimes did not match the experimentally determined flow regimes. Despite these misclassifications, the model demonstrated a strong performance overall, particularly with the 2 and 3 flow regime cases, where it achieved accuracy rates of 83% for both. However, as the number of flow regimes increased, the model’s performance declined. The accuracy dropped to 67% for the 4-flow regime case and further to 58% for the 5-flow regime case, as more misclassifications appeared in the predictions. These results suggest that while the RF model performs well in simpler classification tasks with fewer flow regimes, its ability to accurately predict becomes challenged as the complexity increases, likely due to the difficulty in distinguishing between more granular flow behaviors.

**Table 9:**
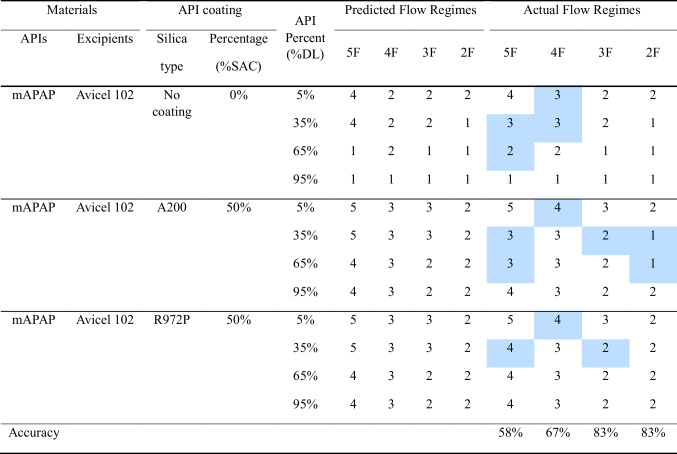
RF Model Predicted and Actual Powder Flow Regimes for Unknown API Percentages, Highlighting Misclassification Cases

#### Unknown API

A separate validation was performed by removing the data for Ibuprofen 25 (IBU25) both individual components (uncoated and dry-coated) and blends, from the training set and reintroducing them into the test set. This approach, as described in Section"Model Validation", aimed to assess the RF model’s performance when faced with an entirely unknown API that has shape and size features not implicitly included in the training data.

The accuracy scores, presented in Table 10, show that the model performs best when predicting 2 flow regimes, achieving a model accuracy of 84.05% and an accuracy of IBU25 prediction of 81%. As the number of categories increased to 3, 4, and 5, the model training accuracy without the IBU25 again remained similar to when it was trained with these data points. The ability to predict the flowability of the unknown IBU25-based powders, however, decreased below the model accuracy. For instance, the model accuracy dropped to 58% for five flow regimes, while the accuracy of predicting the IBU25 powders fell to 46%These results align with trends observed in previous sections, demonstrating the strong predictive capabilities of the RF model for entirely unknown API. This reinforces the RF model's general reliability in lower complexity regimes, with performance declining as the number of flow regimes increases.

**Table 10:** Predicted and Actual powder Flow Regimes for Unknown API Constituent

Number of Flow Regimes	Model Accuracy Training Without IBU25 (RF Model)	Accuracy in Predicting IBU25 (Experiment)	
		Accuracy Score	Correct Predicted Cases (out of 59 IBU25 data)
2	84.05%	81.36%	48
3	73.50%	50.85%	30
4	68.66%	52.54%	31
5	57.83%	45.76%	27

## Conclusion

Six machine learning models (Random Forest, XGBoost, Support Vector Machine, Logistic Regression, K-Nearest Neighbors, and Multi-Layer Perceptron) were evaluated for their ability to predict powder flow regimes in individual components and blends, using 2, 3, 4, and 5 flow regimes. Among the models, the RF model emerged as the most accurate for the 2-flow regime case, achieving prediction accuracies of 85% for individual components and 84% for blends. A comparison of prediction accuracies between individual components and blends revealed generally comparable performance, though accuracies declined for both as the number of flow categories increased. In most scenarios, individual components showed slightly higher prediction accuracies than blends. An exception was observed in the uncoated scenario with 2 flow regimes, where blends achieved a higher accuracy of 94.67% compared to 88.89% for individual components. These results highlight the efficacy of the RF model in handling the complexity of powder flow behavior. The implementation of the ML models in this study allows for the accurate prediction of flow regimes using key material properties such as size, density, surface energy, and particle shape. This predictive capability can be crucial for early-stage decision-making during material selection and help reduce the need for excessive experimental analysis, leading to more efficient, material-sparing processes.

The impact of dry coating on model performance was assessed. Results showed that classification accuracy was highest for uncoated powders, with the RF model achieving 88.89% accuracy for individual components and 94.67% for blends in the 2-flow regime case. The introduction of dry-coated powders led to a decline in accuracy, likely due to modifications in powder properties that increased classification complexity. However, the overall trends remained consistent, with models performing best when fewer flow regimes were considered. The observed differences in performance highlight the influence of surface modifications on flowability predictions and suggest that additional considerations, such as powder properties and adhesion effects, are necessary to enhance model robustness when predicting flow regimes for dry-coated systems. This study underscores the importance of integrating particle engineering approaches that explicitly account for coating-induced changes in powder behavior to improve predictive performance.

SHAP and Feature Importance analysis from the RF model further revealed that critical parameters for predicting flow regimes of blends are the API percentage (%DL), coating percentage (%SAC), and the type of silica used. These insights contribute to a better understanding of the factors that influence flow behavior, particularly in complex blends. When compared to experimental results, the RF model demonstrated high accuracy in predicting flow regimes, with prediction accuracies of 83% for 2 and 3 flow regimes. However, accuracy dropped to 67% and 58% for 4 and 5 flow regimes, respectively, indicating that the model's performance decreases with increasing flow regime complexity. This emphasizes the need for further model refinement, particularly in handling higher flow regime classifications to reduce misclassifications.

Furthermore, the RF model was validated using a leave-one-API-out strategy, where an API was excluded during training and tested separately. The model maintained comparable accuracy to experimental results, demonstrating its robustness in predicting powder flow behavior. These findings highlight the potential of machine learning to enhance the prediction of complex material properties in pharmaceutical formulations.

## Data Availability

Data will be made available on request.
